# Draft Genome Sequence of *Lactobacillus casei* DPC6800, an Isolate with the Potential to Diversify Flavor in Cheese

**DOI:** 10.1128/genomeA.00063-16

**Published:** 2016-03-03

**Authors:** Ewelina Stefanovic, Aidan Casey, Paul Cotter, Daniel Cavanagh, Gerald Fitzgerald, Olivia McAuliffe

**Affiliations:** a Teagasc Food Research Centre, Moorepark, Fermoy, Co. Cork, Ireland; b School of Microbiology, University College Cork, Cork, Ireland

## Abstract

*Lactobacillus casei* is a nonstarter lactic acid bacterium commonly present in various types of cheeses. It is believed that strains of this species have a significant impact on the development of cheese flavor. The draft genome sequence of *L. casei* DPC6800, isolated from a semi-hard Dutch cheese, is reported.

## GENOME ANNOUNCEMENT

*Lactobacillus casei* is a member of the lactic acid bacteria, a group of Gram-positive, facultatively anaerobic and fastidious bacteria with many biotechnological and health-related applications (1). Strains of the *L. casei* species show extraordinary niche adaptability and have been found in various habitats, such as milk and dairy products, plant materials, and in the human and animal gastrointestinal tracts (1, 2). In dairy products, this organism forms part of the nonstarter microbial flora, which has a prominent role during cheese ripening in the development of specific flavor and aroma compounds (3) through the breakdown of numerous substrates, such as amino acids, fatty acids, and carbohydrates, during cheese production and ripening (4). The subject of this analysis, *L. casei* DPC6800, was isolated from a semi-hard Dutch cheese.

Bacterial DNA from strain DPC6800 was extracted, and single-end sequencing was performed on a Roche 454 FLX sequencer housed in the Teagasc Sequencing Centre (Moorepark, Fermoy, Cork, Ireland) using standard protocols from the manufacturer (Roche Diagnostics Ltd., West Sussex, United Kingdom). Quality filtering, adapter clipping, and trimming of the resulting reads as well as assembly were performed using the SeqMan NGen application of the DNAStar Lasergene Genomics Suite (DNASTAR Inc., Madison). Open reading frames (ORFs) were predicted using Glimmer v3.02 (5) and RAST (6). The genome was annotated using the RAST server, with subsequent annotations verified and manually curated using BLASTp (7) and Artemis (8).

Sequence assembly yielded a 3,053,365 bp draft genome with 31× average coverage, consisting of 58 nonoverlapping contigs with a contig *N*_50_ of 98,006 bp and a maximum contig size of 595,092 bp. Whole-genome annotation determined that strain DPC6800 contained a total of 3,300 protein-coding genes and 14 tRNAs. Genes that encode enzymes of crucial importance for flavor development were identified, including components of the proteolytic system such as proteinases, peptidases, and aminotransferases. The cell-wall associated proteinase PrtP (AC564_0739c) was identified, along with numerous peptidases of broad or specific peptidolytic function, such as tripeptide aminopeptidase (AC564_0751c), methionine aminopeptidase (AC564_0890), aminopeptidase S (AC564_0896), aminopeptidase N (AC564_1879c), aminopeptidase V (AC564_3148c), aminopeptidase C (AC564_3291, AC564_3292), and Xaa–Pro-dipeptidyl peptidase (AC564_2630, AC564_2631). Aminotransferases, responsible for the interconversion of amino acids in the later steps of the proteolytic process, are encoded by several genes, i.e., three aspartate aminotransferases (AC564_0742c, AC564_2175, AC564_2467c), two aromatic amino acid aminotransferases (AC564_1682c, AC564_3204), and one branched-chain amino acid aminotransferase (AC564_2001). A gene for glutamate dehydrogenase (AC564_0811c), an enzyme that supports aminotransferase activity through recycling of α-ketoglutarate, an intermediate molecule in aminotransferase reactions, was also identified. Also important for flavor development is the metabolism of citrate, and the presence of a gene encoding an Mg2+-citrate co-transporter CitMHS, necessary for the initial steps of citrate metabolism, was confirmed (AC564_1305). The findings of the genome analysis confirm the potential of *L. casei* DPC6800 for use as an adjunct culture in cheese production to direct or enhance cheese flavor.

### Nucleotide sequence accession numbers.

This whole-genome shotgun project has been deposited at DDBJ/EMBL/GenBank under the accession number LNQD00000000. The version described in this paper is version LNQD01000000.
